# The Role of Serotonin in Brain Development: From Molecular Pathways to Neurodevelopmental Risk

**DOI:** 10.1007/s10571-026-01746-2

**Published:** 2026-05-18

**Authors:** Wing Ki Chan, Sofia Rasmusson, Seyedeh Marziyeh Jabbari Shiadeh, Rebecka Wilhelmsson, Maria E. Johansson, Betina Elfving, Heidi Kaastrup Müller, Carina Mallard, Maryam Ardalan

**Affiliations:** 1 Department of Physiology, Institute of Neuroscience and Physiology, Sahlgrenska Academy, University of Gothenburg, Medicinaregatan 11, Box 432, Gothenburg, 40530 Sweden; 2 Department of Clinical Medicine, Translational Neuropsychiatry Unit, Aarhus University, Palle Juul-Jensens Boulevard 11, 8200 Aarhus N, Denmark

**Keywords:** Autism, Neurodevelopmental disorder, 5-HT, Tryptophan

## Abstract

Serotonin (5-hydroxytryptamine, 5-HT) is a multifaceted neuromodulator involved in processes from early embryogenesis to postnatal brain maturation. During pregnancy, 5-HT plays critical roles not only in maternal mood and behavior but also in placental function and fetal brain development. Beyond its classical role in mood regulation, 5-HT acts as a developmental signal influencing neuronal proliferation, migration, and synaptic organization within precisely timed critical windows. This narrative review provides an integrative framework linking molecular, cellular, and systems-level 5-HT mechanisms to neurodevelopmental outcomes, emphasizing how maternal, placental, and fetal sources of 5-HT dynamically interact with genetic, environmental, and pharmacological factors during gestation. By considering recent advances from developmental neurobiology, genetics, and psychopharmacology, we propose that disrupted 5-HTergic homeostasis represents a convergent mechanism underlying neurodevelopmental risk, particularly in autism spectrum disorder (ASD). We further highlight emerging evidence for sex-dependent 5-HTergic signaling and its role in differential susceptibility across offspring. These results collectively indicate the key role of 5-HT in neurodevelopment and highlight its promise as a biomarker and therapeutic target in neurodevelopmental disorders.

## Introduction

The human brain develops and functions through a complex interplay of molecular, genetic, and environmental factors. Among these, 5-HT has emerged as a key regulator of early neurodevelopment. Primarily produced in the brain and gastrointestinal tract (Pourhamzeh et al., 2022), 5-HT modulates critical processes from embryonic brain formation to postnatal maturation. In the central nervous system (CNS), 5-HTergic neurons are among the earliest to mature (Lidov & Molliver, 1982). Despite the relatively low number of 5-HTergic neurons relative to the overall number of neurons in the CNS, 5-HTergic innervation is dense and widespread across various regions of the brain and spinal cord (Gaspar, Cases, & Maroteaux, 2003), further signifying the important role of 5-HT in early neurodevelopmental processes. Notably, the prefrontal cortex (PFC) and hippocampus are among the major targets of 5-HTergic modulation. Both regions are critically involved in higher-order cognitive, emotional, and social processes and are highly sensitive to alterations in 5-HT signaling during development, making them particularly relevant to the pathophysiology of neurodevelopmental disorders (Lambe, Fillman, Webster, & Shannon Weickert, 2011).

Disruptions to 5-HTergic signaling during sensitive periods of early brain development are associated with an increased risk of neurodevelopmental disorders (NDDs), such as Autism Spectrum Disorder (ASD), Fragile X Syndrome (FXS), and Attention Deficit Hyperactivity Disorder (ADHD). NDDs represent a diverse group of conditions with complex and multifactorial etiologies that arise from interactions between genetic susceptibility and environmental exposures. The frequent overlap in symptoms and the high rate of comorbidity among NDDs pose significant challenges for accurate diagnosis and effective intervention.

Despite the childhood onset of NDDs, these conditions have long-term effects that persist into adulthood, resulting in significant long-term challenges in various aspects of life, such as difficulties in learning, communication, and social functioning, for both affected individuals and their families. The significant, lifelong impact of NDDs highlights the urgent need to enhance our comprehension of the underlying mechanisms and to develop effective therapeutic strategies.

In this narrative review, we discuss recent advances in our understanding of the diverse roles of 5-HT in neuronal function and examine how disruptions in 5-HTergic signaling may contribute to the development and progression of the various neurodevelopmental disorders.

## Serotonin: A Key Neurotransmitter in Diverse Neuronal Functions

**Fig. 1:**
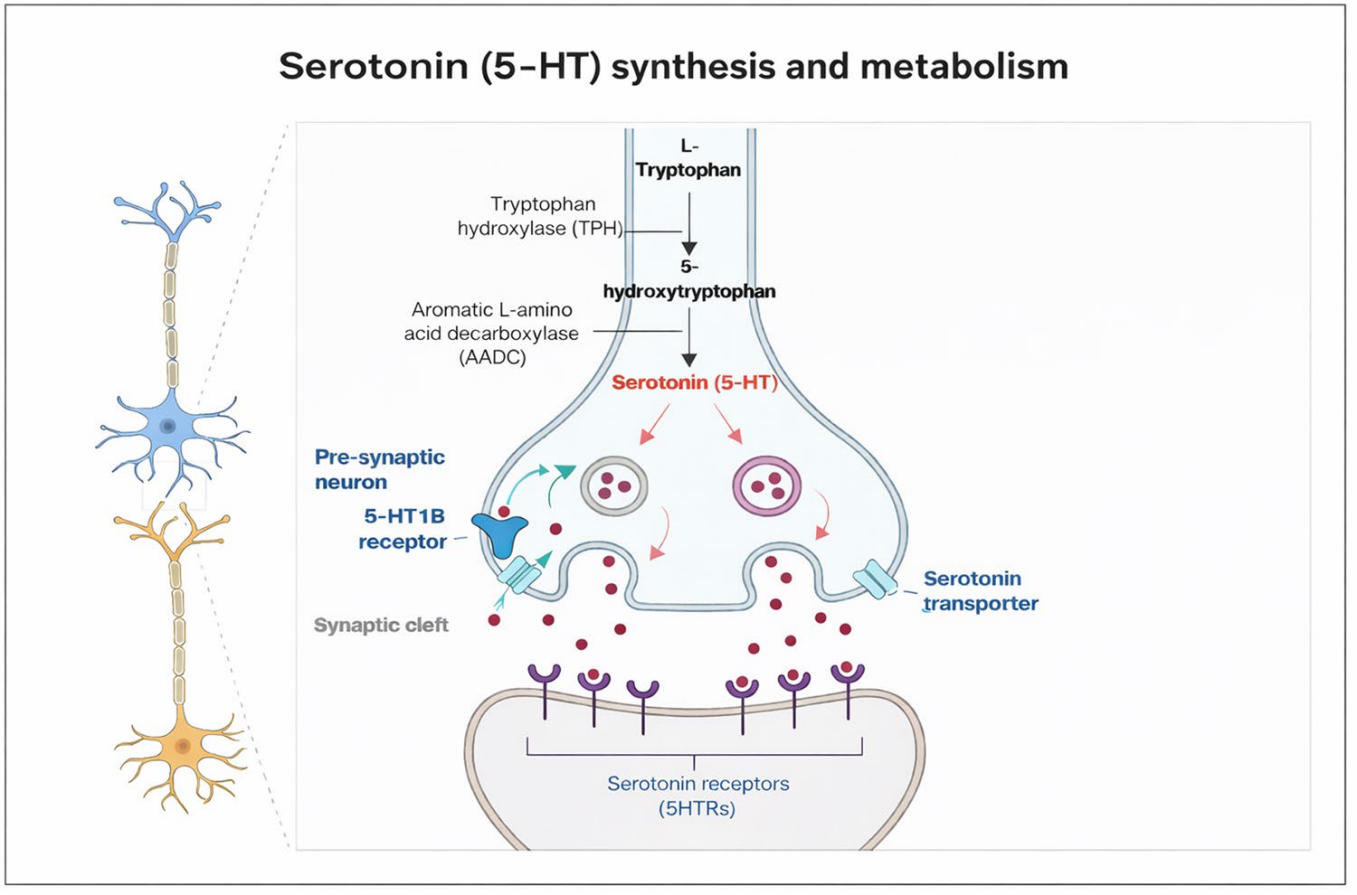
5-HT is synthesized from L-tryptophan, an amino acid, which is converted to 5-HTP by the enzyme TPH. The newly formed 5-HTP then undergoes carboxylation by the enzyme AADC, which transforms 5-HTP into 5-HT (Maffei, 2020). 5-HT is then transported by vesicles and released into the synaptic cleft following an action potential. The 5-HT present in the synaptic cleft can bind to both pre- and post-synaptic 5-HTRs. SERTs present in the pre-synaptic membrane remove and recycle free-5-HT from the synaptic cleft into the pre-synaptic axon terminals, regulating and controlling the actions of 5-HT (5-HT: serotonin; 5-HTP: 5-hydroxytryptophan; TPH: tryptophan hydroxylase type 2; AADC: 1-aromatic acid decarboxylase; 5-HTRs: 5-HT receptors; SERT: 5-HT reuptake transporter). Created in BioRender

5-HT is a monoamine neurotransmitter involved in multiple physiological and behavioral processes, including mood, memory, learning, cognition, sleep regulation, and reward processing (John Jayakumar & Panicker, 2021; Leiser et al., 2015). The synthesis and metabolism of 5-HT involve a series of well-defined steps, as illustrated in Fig. 1. In the body, approximately 90% of total 5-HT is produced in the peripheral nervous system (PNS), primarily in the gastrointestinal tract, where it is synthesized by enterochromaffin cells and enteric neurons. In contrast, only about 10% of 5-HT is synthesized within the central nervous system (CNS), where it is produced by 5-HTergic neurons located in the dorsal and median raphe nuclei of the brainstem (Guzel & Mirowska-Guzel, 2022). 5-HT synthesis in the central and peripheral systems is mediated by two distinct isoforms of the rate-limiting enzyme tryptophan hydroxylase (TPH): TPH1, which is primarily expressed in peripheral tissues, and TPH2, which is mostly expressed in 5-HTergic neurons of the brainstem. Such a distinction has been demonstrated by a study showing that deletion of TPH1 abolishes peripheral 5-HT synthesis while largely preserving central 5-HT levels (Walther et al., 2003). Traditionally, 5-HT has been considered unable to cross the blood-brain barrier (BBB), and central and peripheral 5-HT signaling have therefore been viewed as functionally independent (Ritzen, Hammarstroem, & Ullberg, 1965; Shu et al., 2025; Yabut et al., 2019). However, some experimental evidence suggests a more complex interaction between these systems without contradicting the barrier function of the BBB. For example, Nakatani and colleagues measured blood and brain 5-HT levels in rats following intravenous administration of 5-hydroxytryptophan (5-HTP), the immediate precursor of 5-HT that, unlike 5-HT itself, can cross the BBB and be converted to 5-HT within the brain. Under these conditions, increases in brain extracellular 5-HT were accompanied by parallel increases in whole-blood 5-HT, an effect abolished by pre-treatment with a selective serotonin reuptake inhibitor (SSRI) despite sustained elevation of brain 5-HT. These findings support the presence of transporter-mediated efflux of 5-HT from the brain to the circulation. Importantly, this unidirectional brain-to-blood transport does not imply that circulating 5-HT can cross the BBB to enter the brain parenchyma, and therefore does not challenge the prevailing view that peripheral 5-HT is excluded from the central nervous system (Nakatani et al., 2008).

**Fig. 2:**
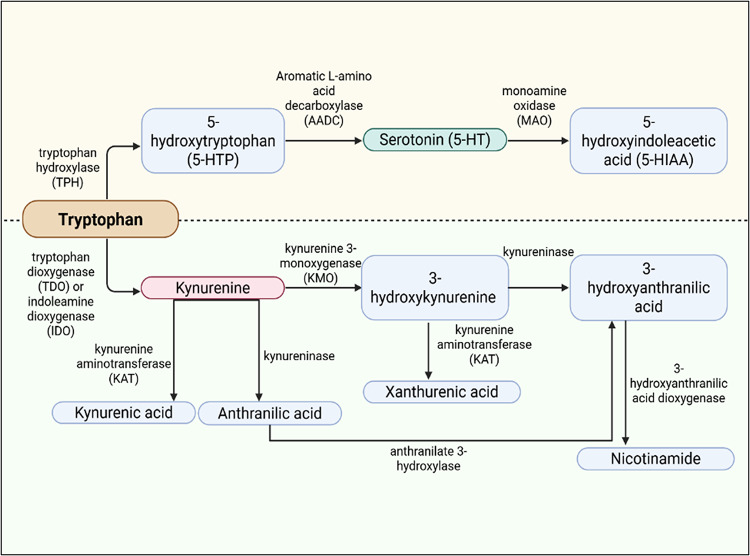
An illustration of the 5-HT and kynurenine pathways of tryptophan metabolism. In the 5-HT pathway (top), tryptophan is converted to 5-HTP by TPH, then to 5-HT by AADC, and subsequently metabolized to 5-HIAA by MAO. In the kynurenine pathway (bottom), tryptophan is converted to kynurenine via the action of TDO or IDO. Kynurenine is further metabolized into kynurenic acid by KAT, anthranilic acid by kynureninase, or 3-hydroxykynurenine by KMO. 3-hydroxykynurenine can be converted to xanthurenic acid by KAT, or to 3-hydroxyanthranilic acid by kynureninase. Anthranilic acid can also be converted to 3-hydroxyanthranilic acid via anthranilate 3-hydroxylase. 3-hydroxyanthranilic acid can be further metabolized to nicotinamide by 3-hydroxyanthranilic acid dioxygenase (Brooks, Mutengwa, Abdalla, Yeoman, & Patel, 2019) (5-HT: serotonin; 5-HTP: 5-hydroxytryptophan; TPH: tryptophan hydroxylase; AADC: aromatic L-amino acid decarboxylase; 5-HIAA: 5-hydroxyindoleacetic acid; MAO: monoamine oxidase; TDO: tryptophan dioxygenase; IDO: indoleamine dioxygenase; KAT: kynurenine aminotransferase; KMO: kynurenine 3-monooxygenase. Created in BioRender

Several pre-clinical studies have demonstrated that oral administration of tryptophan and 5-hydroxytryptophan increases brain 5-HT levels (Leathwood & Fernstrom, 1990; Lynn-Bullock, Welshhans, Pallas, & Katz, 2004). Unlike 5-HT, both tryptophan and 5-hydroxytryptophan can cross the BBB, allowing peripheral modulation to influence central 5-HT synthesis (Turner, Loftis, & Blackwell, 2006; Yabut et al., 2019). Besides serving as a precursor for 5-HT biosynthesis, more than 95% of ingested tryptophan is metabolized via the kynurenine pathway, generating a series of neuroactive metabolites that can modulate neuronal excitability and neurotransmission, including kynurenine (Davis & Liu, 2015; Muneer, 2020). A detailed discussion of the kynurenine pathway is beyond the scope of the present review, but is noted here due to its potential influence on brain 5-HT dynamics (Fig. 2).

Serotonin exerts its diverse developmental effects through multiple receptor subtypes that can be broadly classified based on their intracellular signaling pathways (Brummelte, Mc Glanaghy, Bonnin, & Oberlander, 2017). Gi/o-coupled receptors, including 5-HT_1A_ and 5-HT_1B_, generally inhibit adenylate cyclase activity and reduce neuronal excitability, and are critically involved in early processes such as axonal guidance, synaptic refinement, and regulation of neuronal activity during development. In adults, they primarily modulate synaptic transmission and emotional learning, with 5-HT_1A_ linked to anxiety and memory via hyperpolarization (Carhart-Harris & Nutt, 2017; Gray, 2007; Stiedl, Pappa, Konradsson-Geuken, & Ogren, 2015).

In contrast, Gq/11-coupled receptors, such as 5-HT_2A_, 5-HT_2B_, and 5-HT_2C_, activate phospholipase C signaling pathways, producing IP3 and DAG for cytoskeletal remodeling, gene expression, and modulation of both neuronal and glial function, including microglial activity during critical postnatal windows. They drive remodeling during development, such as 5-HT_2B_ modulating microglial process dynamics and synaptic pruning in postnatal stages like thalamic refinement. Adult roles focus on glial modulation, neuronal plasticity, and neuroinflammation (Kolodziejczak et al., 2015; Zheng & Xu, 2025).

Gs-coupled receptors, including 5-HT4, 5-HT6, and 5-HT7, stimulate cAMP production and are associated with neuronal differentiation, synaptic plasticity, and cognitive processes, particularly during later developmental stages and in the mature brain. For example, in later development and postnatal periods, 5-HT4 supports neurogenesis and neuronal survival (e.g., enteric neurons), 5-HT6 aids hippocampal plasticity during the third postnatal week, and 5-HT7 enhances neurite outgrowth, spine density, and synaptic contacts via Rho GTPases. By adulthood, they regulate transmission (e.g., glutamate release), LTP/LTD, cognition, and mood, with expression peaking in childhood before stabilizing (Brummelte et al., 2017; Ciranna & Catania, 2014; Lambe et al., 2011; Stiedl et al., 2015).

While many of these receptors continue to function in the adult brain, their roles shift from primarily guiding circuit formation during development to modulating synaptic transmission, plasticity, and behavior in mature neural networks (Table 1).

**Table 1:** Classification of serotonin (5-HT) receptor subtypes, signaling pathways, and functional roles in brain development and adulthood

Subtype Group	Key Developmental Roles	Key Adult Roles
Gi/o (e.g., 5-HT_1A/B_)	Axonal guidance, refinement (Gray, 2007)	Synaptic inhibition, emotional memory (Stiedl et al., 2015)
Gq/11 (e.g., 5-HT_2A/B/C_)	Cytoskeletal/microglial changes (Kolodziejczak et al., 2015)	Glial modulation, plasticity (Zheng & Xu, 2025)
Gs (e.g., 5-HT_4/6/7_)	Differentiation, outgrowth (Liu, Kuan, Wang, Hen, & Gershon, 2009)	Transmission, LTP/cognition (Stiedl et al., 2015)

These receptor families differ in both their anatomical distribution and functional roles, enabling 5-HT to regulate a wide range of brain functions (Barnes & Sharp, 1999; Hoyer et al., 1994). The concentration of 5-HT in the synaptic cleft is tightly regulated by 5-HT reuptake transporters (SERTs), which remove extracellular 5-HT, terminating its actions and recycling it into pre-synaptic terminals (Aggarwal & Mortensen, 2017). By controlling the extent and duration of 5-HTergic signaling, SERT plays a critical role in neurotransmission and is a key therapeutic target in psychiatric disorders associated with reduced synaptic 5-HT levels, such as Major Depression disorder (Pourhamzeh et al., 2022).

Originating from the dorsal and medial raphe nuclei, 5-HTergic innervation is spread across numerous brain regions, with particularly dense projections being found in the prefrontal cortex. 5-HT exerts various effects on cortical neuronal activity through 5-HT receptors, for instance, by hyperpolarizing pyramidal neurons via 5-HT_1A_ receptor activation or depolarizing them via 5-HT_2A_ receptor activation. The effects of 5-HT are specific to cell type and projection type. For example, 5-HT suppresses corticopontine neurons while activating callosal/commissural neurons in the medial prefrontal cortex, indicating precise circuit-level modulation (Celada, Puig, & Artigas, 2013). Overall, these effects highlight the important role of 5-HT in shaping cortical activity.

The role of 5-HT in regulating brain function and neuronal plasticity has been well established (Higa et al., 2022). 5-HTergic neurons are highly adaptable and responsive to both hormonal and environmental signals, enabling them to modify their structure and output accordingly. 5-HT receptors have been implicated in modulating astrocyte-derived proteins involved in promoting the growth of 5-HTergic neurons (Whitaker-Azmitia, Murphy, & Azmitia, 1990). Notably, a feedback mechanism has been described in which 5-HTergic projections not only transmit signals to target regions but also receive regulatory input from these areas back to the raphe nuclei. This bidirectional communication further highlights the dynamic regulatory role of the 5-HTergic system. Collectively, these features underscore the importance of 5-HT in supporting neural tissue stability and adaptability, thereby contributing to overall brain function and plasticity (Azmitia, 1999).

## Serotonin and Brain Development

### Maternal and Placental Sources of Serotonin

During early development, before the maturation of the fetal 5-HTergic system, maternal and placental sources contribute significantly to 5-HTergic signalling in the developing brain. The precise origin of maternally derived 5-HT remains debated, with both the placenta and maternal circulation proposed as potential sources. Ex vivo studies demonstrate that the placenta can synthesize 5-HT efficiently, with newly produced 5-HT detectable in the embryonic umbilical vein following maternal tryptophan administration via the uterine artery (Bonnin et al., 2011).

A recent study using a dually perfused human placenta demonstrated that the placenta actively clears fetal serotonin (5-HT) via Organic Cation Transporter 3 (OCT3), metabolizes it to 5-hydroxyindoleacetic acid (5-HIAA) through Monoamine oxidase A (MAO-A), and transports 5-HIAA to the maternal circulation via Multidrug Resistance-associated Protein 2 (MRP2). These findings highlight that placental serotonin clearance in addition to its production not only its production is essential for maintaining 5-HTergic balance in the fetoplacental unit and may be influenced by external factors such as antidepressant exposure (Staud et al., 2023).

Placental 5-HT also exerts epigenetic effects, as it can be covalently attached to histone H3 in a process known as histone serotonylation, dynamically regulating gene expression during placental development and influencing pathways relevant to metabolism and neurodevelopment. Deletion of the SERT has been shown to reduce H3 serotonylation across the placental genome and disrupt neurodevelopmental gene networks in embryonic brain regions (Chan et al., 2024).

On the other hand, some studies question the capacity of the placenta to produce 5-HT. An early mouse embryo study showed that 5-HT immunoreactivity was localized to the ectoplacental cone and placental giant cells, increased following the addition of exogenous 5-HT, and was markedly reduced by the SSRI fluoxetine or by culturing the conceptuses in 5-HT-depleted serum. These findings suggest that placental 5-HT derives from uptake rather than local synthesis (Yavarone, Shuey, Sadler, & Lauder, 1993). Subsequent studies further supported this conclusion, reporting an absence of tryptophan hydroxylase (TPH) expression in trophoblastic tissues of human and mouse placentas, indicating that 5-HT may instead be transferred from maternal blood to the fetus via SERT or through gap junctions (Kliman et al., 2018). More recently, maternal serotonin has been shown to be actively transported into human cytotrophoblasts via SERT, where it accumulates in the nucleus and promotes histone H3 glutamine-5 serotonylation. This epigenetic modification regulates gene expression and cytotrophoblast differentiation independently of local serotonin synthesis (Morris et al., 2025).

### Serotonin and Embryonic Brain Development

Following the contribution of maternal and placental sources, the fetal 5-HTergic system begins to develop early during embryogenesis. In humans, 5-HT is detectable in the medulla oblongata, pons, and spinal cord as early as gestational weeks 5 to 6 (Sundstrom et al., 1993), suggesting a role for serotonin in the earliest stages of brain development. 5-HTergic neurons begin forming in the hindbrain and project rostrally toward the forebrain, with axonal extensions reaching the cerebral cortex by approximately gestational week 10 and the most rostral regions by week 13 (Zecevic & Verney, 1995). By the beginning of the second trimester, around gestational week 15, 5-HTergic cell bodies cluster to form the raphe nuclei, the primary sites of 5-HT synthesis in the CNS (Hanswijk et al., 2020).

During embryonic development, 5-HT plays a crucial role in key neurodevelopmental processes, including cell proliferation, neuronal migration, and axonal wiring (Bonnin et al., 2011; Brezun & Daszuta, 1999; Hanswijk et al., 2020; Vitalis, Ansorge, & Dayer, 2013). Evidence from mouse models lacking tryptophan hydroxylase-1 (TPH-1) and tryptophan hydroxylase-2 (TPH-2), the key enzymes responsible for 5-HT synthesis in the PNS and CNS, respectively, has shown delayed maturation of the upper cortical layers and reduced body weight (Alenina et al., 2009; Cote et al., 2007; Narboux-Neme et al., 2013).

In embryonic mouse studies, 5-HT was shown to inhibit cortical interneuron migration in a dose-dependent and reversible manner. Correspondingly, serotonin transporter (SERT; Slc6a4) knockout mice, which exhibit elevated extracellular 5-HT levels, display abnormal interneuron distribution. Mechanistically, cortical interneurons express 5-HT_6_ receptors, and their activation inhibits interneuron migration, whereas pharmacological blockade reverses this effect (Riccio et al., 2009). These findings indicate that excessive 5-HTergic signalling can disrupt interneuron positioning during cortical development.

In both humans and rodents, thalamocortical axons begin to establish somatosensory maps during the embryonic stage. 5-HT is critically involved in axonal guidance during this period, particularly in shaping thalamocortical projections necessary for relaying sensory input to specific regions of the neocortex (Sinclair-Wilson et al., 2023). 5-HT has been shown to influence the sensitivity of thalamocortical axons to netrin-1, a key axon guidance protein involved in the processes of axon guidance, cell migration, as well as morphogenesis during embryogenesis. This effect is mediated through activation of 5-HT_1B_ and 5-HT_1D_ receptors, highlighting the importance of 5-HT signaling in directing axons to their proper cortical targets and establishing functional neural circuits (Bonnin, Torii, Wang, Rakic, & Levitt, 2007; Bradford, Cole, & Cooper, 2009; Sinclair-Wilson et al., 2023).

### Serotonin and Postnatal Brain Development

As described in the previous section, 5-HT influences neurodevelopment from the earliest stages of life, beginning prenatally and continuing into the postnatal period, during which a certain level of plasticity in the formation of neural circuits is preserved. Multiple 5-HT receptor subtypes, as well as SERT, have been implicated in shaping the postnatal brain.

Among various 5-HT receptor subtypes, ionotropic 5-HT_3_ receptors play a crucial role in modulating GABAergic interneurons, which are essential for controlling neuronal excitability during this period (Engel, Smidt, & van Hooft, 2013). In addition to its neuronal effects, 5-HT also modulates the activity of microglia, the brain’s primary immune cells. Apart from 5-HT_3_ receptors, conditional invalidation of microglial 5-HT_2B_ receptors, the predominant 5-HTergic receptor subtype expressed on microglia, during early postnatal development (between birth and postnatal day 30) was found to disrupt neural circuit formation. This resulted in behavioral abnormalities in adulthood, including hyperactivity and deficits in social interaction and flexibility. Importantly, these outcomes were dependent on the timing of gene invalidation, as later deletion (after P30) did not produce such effects, highlighting a critical window during which microglial 5-HT_2B_ receptors influence the development of social and adaptive behaviors and may contribute to neurodevelopmental disorders such as ASD (Albertini et al., 2023).

Early-life dietary exposures and gut microbiota composition can shape postnatal 5-HT signaling, influencing brain development and neurodevelopmental outcomes. In a study on postnatal rats, maternal dietary modifications, such as caloric restriction or high-fat/high-fructose intake, differentially affect brain 5-HT levels and SERT expression in offspring, with notable sex-specific effects. For example, high-fat/high-fructose exposure reduced brain 5-HT by ~ 32% in male pups at postnatal day 2, while females were unaffected, whereas by postnatal day 21, females exhibited a ~ 40% reduction in brain 5-HT, with only minor changes in males. Intrauterine growth-restricted pups reared on the same diet showed elevated 5-HT, accompanied by a ~ 28% reduction in SERT expression in females, highlighting complex interactions between early-life environment, sex, and 5-HTergic signaling (Ye et al., 2024). These alterations were closely associated with shifts in gut microbiota, suggesting that early-life gut dysbiosis may modulate central 5-HT function. Supporting this link, clinical studies in children with ASD have revealed age-dependent gut microbiome dysbiosis, including decreased Firmicutes/Bacteroidetes and Actinobacteria/Proteobacteria ratios, particularly in early childhood, indicating that disruptions in the gut microbiota may contribute to neurodevelopmental outcomes (Kadiyska et al., 2025). These findings underscore that early-life diet and gut-brain interactions, through effects on 5-HT signaling in postnatal development, may influence neurodevelopment and vulnerability to neuropsychiatric disorders later in life.

Following prenatal development, 5-HT remains a critical regulator of thalamocortical axon maturation during the early postnatal period. In rodents, initial studies demonstrated that 5-HT influences thalamocortical axon activity and somatosensory cortex development through receptor-specific mechanisms, including trophic-like effects mediated by 5-HT_1B_ and 5-HT_2A_ receptors (Mansour-Robaey, Mechawar, Radja, Beaulieu, & Descarries, 1998). Mechanistic insight was further provided by studies in monoamine oxidase A knockout (MAOA-KO) mice, in which excess postnatal 5-HT disrupted activity-dependent refinement of thalamocortical axons in layer IV, preventing barrel formation through impaired axonal branching and collateral retraction. Notably, deletion of the presynaptic 5-HT_1B_ receptor rescued this phenotype, highlighting a direct role for 5-HTergic signaling in axon refinement (Rebsam, Seif, & Gaspar, 2002). Subsequent work in juvenile serotonin transporter (SERT) knockout rats demonstrated that excessive 5-HT also alters barrel field architecture, reduces intracolumnar organization of layer IV neurons, and impairs the precision of thalamocortical projections, leading to abnormal recruitment of layer Vb into intracortical circuits (Miceli et al., 2013). Although these studies primarily focus on sensory thalamocortical circuits, they reveal fundamental mechanisms by which 5-HT regulates axonal refinement during early postnatal development. To date, most mechanistic evidence on 5-HTergic modulation of thalamocortical maturation comes from rodent models. Importantly, human fetal studies suggest that similar 5-HTergic mechanisms may operate prenatally. Immunohistochemical analyses of fetuses between 21 and 32 weeks of gestation reveal early and dynamic expression of multiple 5-HT receptor subtypes, including 5-HT_2A_, 5-HT_2C_, 5-HT_1A_, and 5-HT_4_, across distinct thalamic nuclei (Wai, Lorke, Kwong, Zhang, & Yew, 2011). The developmental increase and spatial expansion of these receptors suggest that 5-HT contributes to thalamic maturation and connectivity in humans, and that disruption of 5-HTergic signaling during this critical period may increase vulnerability to neuropsychiatric disorders. Together, these findings suggest that early 5-HT imbalance could induce widespread disruption in intracortical connectivity during early postnatal development.

The identification of inhibitory 5-HT autoreceptors on raphe neurons led to the proposal that 5-HT regulates the development of its own circuitry through autocrine mechanisms (Teissier, Soiza-Reilly, & Gaspar, 2017). Consistent with this idea, experimental manipulations of brain 5-HT levels during development have repeatedly shown that 5-HT availability influences raphe neuron connectivity. However, these effects are highly dependent on the experimental model, brain region examined, and developmental timing, resulting in non-uniform outcomes rather than a single, global pattern of growth regulation. For example, Migliarini et al. selectively altered central 5-HT levels during development and reported pronounced region-specific changes in 5-HTergic innervation: reduced 5-HT projections to the suprachiasmatic and thalamic paraventricular nuclei, contrasted with excessive 5-HTergic fiber density in the nucleus accumbens and hippocampus. These findings indicate that altered 5-HT signaling does not uniformly promote or inhibit 5-HTergic growth, but instead differentially sculpts target-specific circuits. Notably, this was accompanied by increased expression of brain-derived neurotrophic factor (BDNF) in the hippocampus, suggesting the engagement of compensatory neurotrophic mechanisms in response to altered 5-HT signaling (Migliarini, Pacini, Pelosi, Lunardi, & Pasqualetti, 2013). Further supporting a developmental role for 5-HT, early postnatal inhibition of 5-HT synthesis using para-chlorophenylalanine (PCPA) led to persistent behavioral alterations, including impaired short-term recognition memory, increased anxiety-like behavior, and heightened pain sensitivity, particularly in females. These behavioral effects were associated with reduced BDNF levels in both the hippocampus and prefrontal cortex (Saadati, Sadegzadeh, Sakhaie, Panahpour, & Sagha, 2021). Together, these studies demonstrate that while 5-HT clearly influences the development of its own circuitry, its effects are context-dependent and mediated, at least in part, through interactions with neurotrophic signaling pathways that shape long-term behavioral outcomes.

In summary, 5-HT plays multifaceted roles in postnatal neurodevelopment, including the regulation of neuronal circuit formation, synaptic plasticity, and region-specific innervation, particularly in the prefrontal cortex and hippocampus. Disruptions in 5-HTergic signaling during these sensitive developmental windows may contribute to long-term alterations in brain function and increase susceptibility to neurodevelopmental disorders.

## The Role of the 5-HTergic System in Neurodevelopmental Disorders

Neurodevelopmental disorders are characterized by distinct but overlapping behavioral phenotypes, including deficits in social interaction and communication in ASD, cognitive impairment and sensory hypersensitivity in FXS, and inattention and hyperactivity in ADHD. These overlapping phenotypes stem from early alterations in neuronal wiring, synaptic plasticity, and circuit maturation (Bagni & Zukin, 2019; de la Pena, Pan, Thai, & Alisso, 2020; Devitt, Gallagher, & Reilly, 2015).

A variety of animal models, including genetic and pharmacological models, have been developed to recapitulate key aspects of these phenotypes and are widely used to investigate underlying neurobiological mechanisms. Genetic models like Fmr1 knockout mice recapitulate FXS’s synaptic and behavioral deficits (Spencer et al., 2011), while BTBR mice model ASD’s social impairments (Meyza et al., 2013). ADHD is studied via spontaneously hypertensive rats (SHR) or dopamine-lesioned models. Pharmacological approaches, such as prenatal valproic acid (VPA) exposure (Nicolini & Fahnestock, 2018), early-life inflammation induce ASD-like traits across species (Ardalan et al., 2022). Dysregulation of the 5-HTergic system, including both the 5-HT and kynurenine pathways, has been implicated in a range of neurodevelopmental disorders, including ASD, FXS, and ADHD (Borcsiczky et al., 1996; Coleman, 1971; Costa et al., 2012; Schain & Freedman, 1961). These models reveal how 5-HT imbalances impair axonal guidance and synaptic refinement, offering insights for targeted therapies. A better understanding of these neurochemical circuits may aid in developing more targeted and effective therapeutic strategies.

### Autism Spectrum Disorder (ASD)

ASD is a neurodevelopmental condition characterized by deficits in social communication and the manifestation of restricted interests and repetitive behaviors (American Psychiatric Association, 2022). Globally, approximately 1 in 31 children is diagnosed with ASD, with prevalence nearly four times higher in boys than girls. However, this sex difference may be influenced in part by diagnostic bias, as emerging evidence suggests that early ASD-related symptoms in infants are more evenly distributed between sexes (Kaufmann et al., 2025; Prevention, 2025; Zeidan et al., 2022). The involvement of 5-HT in ASD pathophysiology was first proposed in 1961, when Schain and Freedman reported elevated whole-blood 5-HT levels in children with infantile autism (Schain & Freedman, 1961). Since then, hyperserotonemia has been reported in approximately 30% of individuals with ASD (Gabriele, Sacco, & Persico, 2014), indicating a disruption in serotonin regulation that may contribute to altered neurodevelopmental processes. Tryptophan and its downstream metabolites have been investigated in ASD, though findings remain inconsistent. In children with ASD, serum levels of kynurenine and kynurenic acid are elevated compared to neurotypical peers, suggesting an upregulation of the kynurenine pathway in early development (Yildirim, Simsek, Cetin, & Dokuyucu, 2023). In contrast, adults with ASD exhibit reduced concentrations of tryptophan and kynurenic acid relative to first-degree relatives and unrelated controls, implying age-dependent dysregulation of tryptophan metabolism (Carpita et al., 2023). At the cellular level, metabolic profiling of lymphoblastoid cells derived from individuals with ASD has demonstrated a reduction in NADH generation when tryptophan is provided as the sole energy source, suggesting impaired production of critical downstream metabolites such as 5-HT and kynurenic acid (Boccuto et al., 2013). These alterations may influence neurotransmission, neuroinflammation, and redox balance, potentially contributing to ASD-related phenotypes. Emerging evidence also implicates the gut microbiota in modulating tryptophan metabolism. Metabolic analysis of fecal samples from individuals with ASD has revealed distinct microbial compositions compared to neurotypical individuals (Pang et al., 2023). These microbiome differences may shift tryptophan metabolism toward neuroactive metabolites, including kynurenine derivatives and serotonin precursors, thereby influencing central nervous system function and behavior. Taken together, these findings highlight a multifaceted disruption of tryptophan metabolism in ASD, spanning systemic, cellular, and microbial levels, with potential implications for both neurodevelopmental trajectories and therapeutic interventions.

Disruptions in 5-HT signaling during pre- and postnatal development have been linked to abnormal hippocampal neurogenesis; for instance, prenatal exposure to lipopolysaccharide (LPS) has been shown to enhance hippocampal SERT expression and reduce hippocampal neurogenesis in adult female rats (Mouihate, Kalakh, AlMutairi, & Alashqar, 2019). Another study demonstrated that early-life dietary tryptophan restriction - resulting in reduced 5-HT availability - lowered 5-HT levels across several brain regions, including the prefrontal cortex, hippocampus, and brainstem, and is associated with increased anxiety- and depression-like behaviors. These behavioral alterations are accompanied by impaired neurogenesis in the dentate gyrus, reduced neuronal activity, and structural deficits in hippocampal neurons, such as dendritic atrophy and decreased spine density (Zhang et al., 2006). ASD is characterized by atypical brain growth trajectories, including early brain overgrowth followed by slowed or arrested development later in childhood, suggesting disruptions in prenatal and early postnatal neurodevelopment (Courchesne, Campbell, & Solso, 2011). Structural alterations in limbic regions, such as the amygdala and hippocampus, are detectable within the first year of life, indicating that ASD-related brain changes emerge during periods of heightened developmental vulnerability (Li et al., 2019). Together, these observations suggest that altered 5-HT signaling at multiple developmental stages may impair hippocampal neurogenesis, contributing to abnormal brain development in ASD. In this context, increasing attention has been directed toward maternal exposure to SSRIs during pregnancy as a potential modifier of fetal 5-HT signaling and risk factor for ASD, despite findings across epidemiological studies remaining mixed. SSRIs readily cross the placenta and have been found in both the amniotic fluid (Hendrick et al., 2003) and in the cord blood. Maternal use of sertraline has been shown to result in moderate fetal exposure, with umbilical cord blood levels influenced by maternal dose and timing of administration, indicating that SSRIs taken during pregnancy can reach the fetus and potentially alter 5-HTergic signaling (Goutallier et al., 2005). This raise concerns that pharmacological disruption of 5-HTergic homeostasis during critical periods of brain development may influence neurodevelopmental trajectories. A population-based case-control study reported that prenatal SSRI exposure was associated with increased susceptibility to ASD and developmental delay in boys, with prenatal SSRI exposure being approximately three times more frequent in boys with ASD compared to those with typical development (Harrington, Lee, Crum, Zimmerman, & Hertz-Picciotto, 2014); however, the number of female cases in this study was limited, precluding reliable conclusions regarding ASD risk in girls following prenatal SSRI exposure. Further supporting this observation, a large population-based cohort study found that use of antidepressants, specifically SSRIs, during the second and/or third trimester was associated with an increased risk of ASD in children (Boukhris, Sheehy, Mottron, & Berard, 2016), highlighting that timing of exposure may be an important factor in modulating ASD risk. In contrast, findings from the Study to Explore Early Development, a large multisite case-control study conducted in the United States, indicate that maternal psychiatric conditions during pregnancy are associated with increased odds of ASD and developmental delays in offspring, regardless of SSRI treatment, and when analyses were restricted to mothers with psychiatric conditions, SSRI use was not independently associated with ASD risk, suggesting that underlying maternal psychopathology, rather than SSRI exposure, may largely account for the observed associations (Ames et al., 2021). Such findings highlight the potential impact of prenatal 5-HTergic modulation on ASD risk and the challenge of separating drug effects from maternal psychiatric factors, emphasizing the importance of considering other factors, such as timing of SSRI exposure and maternal context, in studies of maternal SSRI exposure. Together, these findings underscore that both prenatal 5-HTergic modulation and genetic variation in 5-HT pathways may contribute to ASD risk, with sex-dependent effects highlighting the complex interplay between maternal, genetic, and environmental factors.

Complementary to evidence on environmental modulation of 5-HT signaling, genetic studies highlight an additional contribution of 5-HT pathway variation to ASD risk. Genetic variation in *SLC6A4*, which encodes the 5-HT transporter, has been linked to ASD, including male-biased transmission at 17q11.2 and rare coding variants associated with increased rigid-compulsive behaviors (Sutcliffe et al., 2005). Importantly, *SLC6A4* variation primarily affects synaptic 5-HT reuptake rather than causing global depletion of serotonin.

Consistent with a gene–environment interaction model, maternal *Slc6a4* heterozygosity combined with prenatal stress have been shown to blunt embryonic gene expression, miRNA, and methylation responses, providing a mechanistic link between maternal 5-HTergic variation and ASD-like outcomes, such as heightened anxiety, repetitive behaviour, abnormal sociability and exaggerated startle responses, along with diminished learning ability in aggression tests (Sjaarda et al., 2017). In contrast to transporter-related alterations, experimental models demonstrate that direct depletion of brain serotonin is sufficient to induce ASD-like phenotypes, as *Tph2* knockout mice, which lack central serotonin synthesis, exhibit deficits in social interaction and communication, increased repetitive and compulsive behaviors, delayed developmental milestones, and reduced neonatal preference for maternal scent (Kane et al., 2012). Notably, these mice also display increased aggressive-like behavior, particularly in females (Mosienko et al., 2012). Similarly, *Tph2* knockout rat model show sex-dependent behavioral response to brain 5-HT depletion, with males showing increased copulation-like behaviors and aggression, and females exhibiting increased compulsivity (Golebiowska et al., 2025).

**Fig. 3:**
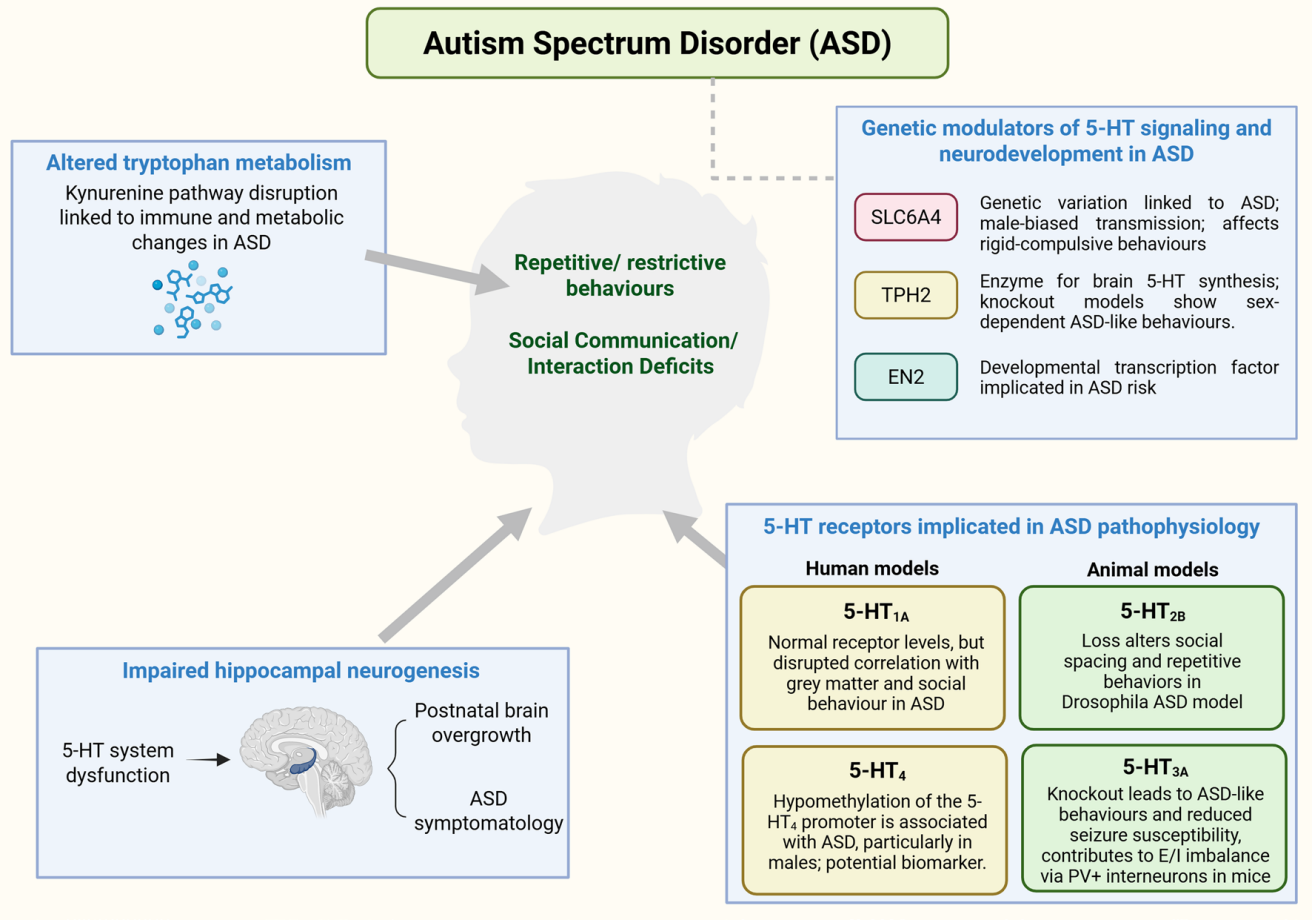
A summary diagram depicting the involvement of the 5-HT system in the pathophysiology of ASD. ASD is characterized by social and communication difficulties, as well as restrictive and repetitive behaviors. Studies have found an association between 5-HT system disruption and the development of an overgrown brain at the postnatal stage, as well as the symptoms observed in individuals with ASD. A link between altered tryptophan metabolism, due to disrupted kynurenine pathway, and the immune and metabolic changes in ASD has been proposed. Genetic variations of the *SLC6A4* gene has been linked to ASD, with male-biased transmission at 17q11.2 and rare coding variants preferentially transmitted in families with increased rigid-compulsive behaviors. *Tph-2* knockout models have been shown to exhibit sex-specific ASD-like behaviors. En2 gene mutations have been found to result in region-specific 5-HT imbalances in animal models, paralleling patterns observed in ASD. Evidence from a post-mortem human study showed that individuals carrying the ASD-associated EN2 haplotype showed increased cerebellar *EN2* mRNA and altered expression of neighboring 5-HT-related genes, linking *EN2* dysregulation to 5-HTergic involvement in ASD. Specific 5-HT receptors, including 5-HT_1A_, 5-HT_2B_, 5-HT_3A_, and 5-HT_4_, have been implicated in ASD pathophysiology through diverse mechanisms affecting neuronal signaling, excitation/inhibition balance, and gene regulation (ASD: Autism Spectrum Disorder; 5-HT: serotonin, *EN2*: Engrailed homeobox 2; *SLC6A4*: solute carrier family 6 member 4 (serotonin transporter); 5-HT_1A_: serotonin receptor 1 A; 5-HT_2B_: serotonin receptor 2B; 5-HT_3A_: serotonin receptor 3 A; 5-HT_4_: serotonin receptor 4). Created in BioRender

Developmental regulators such as the Engrailed-2 (EN2) protein have been implicated in the maturation of the 5-HTergic system in specific subgroups of ASD associated with EN2 genetic variation. In mice, knockout of the engrailed 2 (En2) gene, a transcription factor linked to ASD susceptibility, produces neuropathological features resembling those reported in ASD, including reduced tyrosine hydroxylase, noradrenaline, and 5-HT levels in the hippocampus and cerebral cortex. High-performance liquid chromatography analysis revealed region- and age-dependent alterations in brain 5-HT levels, with reductions in the frontal and occipital cortex at 1 and 3 months and a partial normalization by 6 months, whereas the cerebellar cortex exhibited an early reduction followed by a sustained increase from 3 months onward. These findings suggest postnatal dysregulation of the 5-HT system rather than uniform 5-HTergic deficits. Consistent with this, a post-mortem study in individuals with autism carrying an ASD-associated EN2 haplotype (*rs1861972-rs1861973 A-C/G-T*) reported increased cerebellar EN2 mRNA expression, along with altered expression of neighboring genes which are co-expressed with EN2 during development, including the 5-HTergic receptor gene HTR5A and Sonic Hedgehog gene, SHH, which is responsible for instructing the production of a protein necessary for embryonic development. Together, these observations suggest that altered EN2 signaling may be associated with atypical 5-HTergic system development and could contribute to specific ASD-related phenotypes within EN2-linked subgroups (Viaggi, Gerace, Pardini, Corsini, & Vaglini, 2015) (Choi et al., 2014) (Fig. 3).

Interestingly, behavioral characteristics of ASD, including social deficits and altered fear memory, have been observed in mouse models lacking 5-HT receptor subtypes such as 5-HT_3A_. Interestingly, 5-HT_3A_ knockout mice also exhibit reduced susceptibility to seizures, suggesting that while specific 5-HT receptor loss may contribute to ASD traits, it could simultaneously offer protection against epilepsy, a condition that frequently co-occurs with ASD (Kondo, Nakamura, Ishida, Yamada, & Shimada, 2013). This duality underscores the complexity of 5-HTergic receptor function.

Research has shown that the genetic profile associated with ASD may disrupt the balance between excitatory and inhibitory synaptic signaling, potentially contributing to altered neural connectivity and function (Rubenstein & Merzenich, 2003). The loss of 5-HT_3_ receptors has been linked to increased NMDA receptor activity and increased excitability of parvalbumin-expressing (PV^+^) interneurons. This results in excessive inhibitory GABAergic input to pyramidal neurons and a reduction in the excitatory/inhibitory ratio, contributing to ASD-related neural dysfunction (Huang et al., 2021). In line with these findings, a study in 15q duplication mice, a mouse model of ASD, demonstrated that alterations in prefrontal cortex microcircuits can shift the excitatory/inhibitory (E/I) balance toward a hyperexcitable state. Specifically, enhanced long-term potentiation (LTP) at glutamatergic synapses onto layer 5 pyramidal neurons has been observed, associated with altered glutamatergic and GABAergic inputs onto fast-spiking interneurons (FSINs). Notably, FSIN excitability in these circuits is modulated by constitutive activation of 5-HT2 receptors, highlighting a key role for 5-HTergic signaling in regulating cortical E/I balance at the microcircuit level (Saitow, Takumi, & Suzuki, 2020).

Specific 5-HT receptors also appear to be associated with behavioral, epigenetic, and neuroanatomical features associated with ASD. Using RNA interference and CRISPR/Cas9 techniques, Cao et al. identified 5-HT_2B_ as a key regulator of social spacing and repetitive behaviors in a Drosophila melanogaster model (Cao, Tang, Liu, Huang, & Xu, 2022). In humans, hypomethylation of the 5-HT_4_ receptor promoter has been linked to ASD in adult males, suggesting its potential as a biomarker for ASD risk (Hu et al., 2020). Neuroimaging studies have shown that in neurotypical men, 5-HT_1A_ receptor binding in the posterior putamen correlates with both grey matter volume and specific social behaviour traits,, whereas this relationship is absent in men with ASD (Lefevre, Richard, Mottolese, Leboyer, & Sirigu, 2020). Although overall 5-HT_1A_ receptor availability does not differ between groups, these findings suggest a disruption in the coupling between 5-HTergic signaling, brain structure, and social behavior in ASD, rather than a direct effect on receptor density.

Despite substantial evidence implicating 5-HTergic dysfunction in ASD, pharmacological interventions such as antipsychotics and SSRI have shown limited efficacy in alleviating core or associated symptoms. Aripiprazole, an antipsychotic approved for managing irritability in children with ASD, has a proposed mechanism involving partial agonism at dopamine D2 and 5-HT_1A_ receptors, coupled with antagonism at 5-HT_2A_ receptors, although the precise pathways underlying its therapeutic effects remain unclear (LeClerc & Easley, 2015). Similarly, fluvoxamine, an SSRI, demonstrates only modest benefits in reducing anxiety and ASD-associated behaviors, including repetitive preoccupations and perseveration, and its use is frequently limited by adverse effects such as agitation and sleep disturbances (Martin, Koenig, Anderson, & Scahill, 2003). These studies highlight the limited therapeutic impact of current 5-HT-targeting pharmacotherapies in ASD and underscore the need for novel interventions that more effectively address both core and associated symptoms.

To summarize, 5-HT emerges as an important neurotransmitter in neurodevelopment, exerting a significant impact on processes such as neurogenesis and maintaining a balance between the excitatory and inhibitory activity in the brain. From early observations of altered peripheral 5-HT levels in individuals with ASD to more recent studies on specific receptor subtypes such as 5-HT_2B_, 5-HT_3A_, 5-HT_4_, and 5-HT_1A_, evidence increasingly points to a multifaceted role of the 5-HT system in ASD. Disruptions in 5-HT signaling have been associated with both structural brain changes and core behavioral symptoms of ASD. These findings highlight the potential of 5-HTergic markers for earlier diagnosis and the development of more focused, receptor-specific interventions (Fig.3).

### Fragile X Syndrome (FXS)

FXS is a neurodevelopmental disorder caused by a trinucleotide expansion of the Fragile X Messenger Ribonucleoprotein 1 (*FMR1*) gene on the X chromosome, primarily affecting men (Hagerman et al., 2009; Hernandez et al., 2009). Individuals with FXS often present with abnormally developed physical features, such as an elongated face, a prominent forehead and jaw, large ears, hyperflexible fingers, and, in post-pubertal males, enlarged testicles. Behavioral traits commonly include excessive shyness, poor eye contact, heightened anxiety, and sensory processing difficulties, along with intellectual disabilities, delayed speech development, and features associated with ASD (Stone, Basit, Shah, & Los, 2025). In several pre-clinical studies, disrupted neuroplasticity, along with an excitatory/inhibitory imbalance, was a key finding in FXS animal models (Belmonte & Bourgeron, 2006; Moy & Nadler, 2008; Rubenstein & Merzenich, 2003). In humans, the Fragile X Mental Retardation Protein (FMRP), produced by the *FMR1* gene, is widely expressed throughout the brain, and its absence during early development, when synaptic connections are dynamically regulated, is particularly disruptive (Kadokura, den Adel, Krauwinkel, Takeshige, & Nishida, 2008).

The roles of various 5-HT receptors in FXS and their potential as therapeutic targets have been explored in several studies. It was demonstrated that activation of the 5-HT_7_ receptor reduces exaggerated metabotropic glutamate receptor-mediated long-term depression (mGluR-LTD) in the hippocampus and normalizes abnormal behaviors in *Fmr1* knockout mice, highlighting 5-HT_7_ as a potential therapeutic target in FXS (Costa et al., 2012). They further showed that 5-HT_7_ receptor agonists LP-211 and BA-10, effectively reversed exaggerated mGluR-LTD in the CA3-CA1 hippocampal synapse of the FXS mice model (Costa, Sardone, Lacivita, Leopoldo, & Ciranna, 2015). Further investigation demonstrated that the enzyme cyclin-dependent kinase 5 (Cdk5) modulates 5-HT_7_ receptor-mediated effects on synaptic plasticity. Inhibition of Cdk5 in wild-type hippocampal neurons enhanced mGluR-LTD to levels seen in *FMR1* knockout mice. Moreover, the inhibition of Cdk5 was found to prevent the 5-HT_7_ receptor-mediated reversal of mGluR-LTD in both wild-type and *FMR1* knockout neurons (Costa, Tempio, Lacivita, Leopoldo, & Ciranna, 2021). This suggests that Cdk5 plays a critical role in mediating the synaptic benefits of 5-HT_7_ receptor activation. In contrast to these findings, Tyagi et al. reported that the antiepileptic effects of the 5-HTergic psychedelic N, N-dipropyltryptamine (DPT) in *FMR1* knockout mice were not mediated by 5-HT_1A_, 5-HT_1B_, 5-HT_2A_, or even pan-5-HT receptor blockade, suggesting a non-5-HTergic mechanism despite DPT’s known 5-HTergic profile. This highlights the complexity of 5-HTergic signaling in FXS and suggests that not all 5-HTergic compounds exert their effects through classical receptor pathways (Tyagi, Saraf, & Canal, 2023).

From a clinical perspective, pharmacological interventions specifically targeting the 5-HTergic system, such as SSRIs, particularly fluoxetine, have been used to alleviate symptoms such as anxiety in individuals with FXS, although activation (manifesting in various ways such as mood changes, restlessness, and aggression) has been reported as a potential effect associated with SSRI use (Hagerman et al., 2009). While preclinical data strongly support 5-HT_7_ receptor modulation as a therapeutic strategy (Costa et al., 2015; Costa et al., 2012; Costa et al., 2021), evidence for receptor-specific interventions in humans is still limited.

**Fig. 4:**
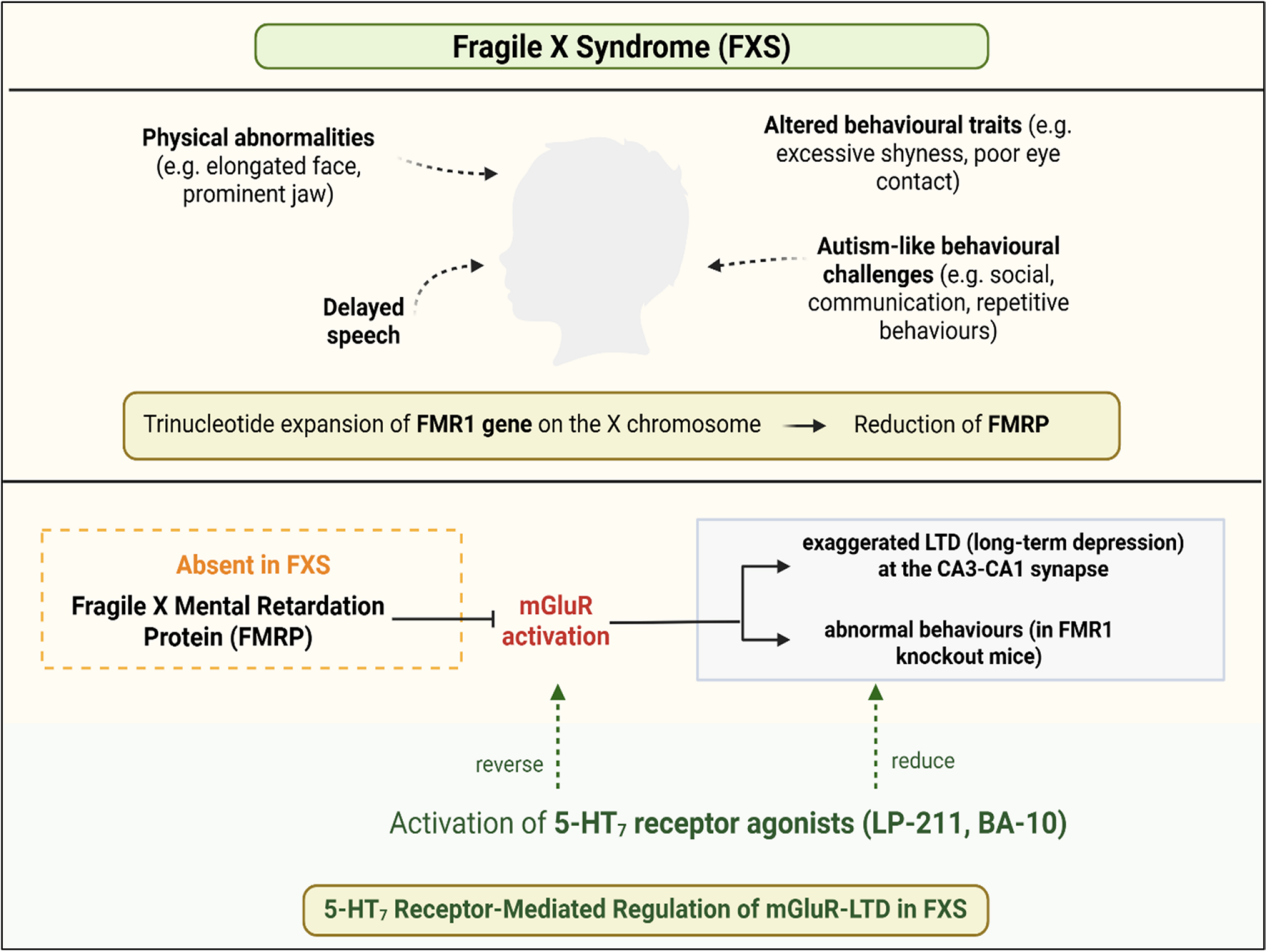
A summary diagram describing the key features of FXS and the possible involvement of the 5-HT_7_ receptor in the pathophysiology of FXS. FXS is characterized by the presence of physical abnormalities, e.g. elongated face and prominent jaw, altered behavioral traits (e.g. excessive shyness, poor eye contact) and ASD-like behavioral challenges (e.g. social and communication difficulties). It is caused by a trinucleotide expansion of the FMR1 gene on the X chromosome, resulting in the absence of the FMRP. Research has found that 5-HT_7_ receptor agonists reduce the exaggerated long-term depression in the hippocampus mediated by mGluR-LTD and abnormal behaviors in FMR1 knockout mice. The involvement of Cdk5 in the role of 5-HT_7_ receptors in modulating hippocampal synaptic plasticity has also been demonstrated, with the inhibition of Cdk5 being shown to prevent the 5-HT_7_ receptor-mediated reversal of mGluR-LTD (FXS: Fragile X Syndrome; 5-HT_7_ receptor: 5-HT receptor 7; ASD: Autism Spectrum Disorder; FMR1: Fragile X Messenger Ribonucleoprotein 1; FMRP: Fragile X Mental Retardation Protein; mGluR-LTD: metabotropic glutamate receptor-mediated long-term depression; Cdk5: cyclin-dependent kinase 5). Created in BioRender

In conclusion, 5-HT signaling, particularly via the 5-HT_7_ receptor, plays a significant role in modulating synaptic dysfunction in FXS. The activation of 5-HT_7_ receptors has been shown to correct exaggerated mGluR-LTD and restore synaptic plasticity, with Cdk5 signaling implicated in this process. However, the observation that compounds like DPT act independently of 5-HT receptors emphasizes the need for further research to delineate 5-HTergic and non-5-HTergic pathways involved in FXS pathophysiology (Fig. 4).

### Attention Deficit Hyperactivity Disorder (ADHD)

ADHD is a common neurodevelopmental disorder characterized by a triad of inattention, hyperactivity, and impulsivity, with behavioral inhibition being regarded as the core deficit (Alderson, Rapport, Hudec, Sarver, & Kofler, 2010; American Psychiatric Association, 2022). While ADHD is often viewed as a childhood disorder, the clinical symptoms of ADHD persist into adulthood in roughly 60% of cases (Kessler et al., 2005). The 5-HTergic hypothesis for ADHD was first proposed by Coleman (1971), who observed reduced serum 5-HT concentrations in children with ADHD (Coleman, 1971). Subsequent studies have supported this hypothesis, reporting similarly lowered 5-HT levels in the blood of children diagnosed with ADHD (Wang et al., 2018). Genetic studies have implicated 5-HTergic mechanisms in ADHD. For instance, one study found a 25% reduction in SERT binding capacity among individuals with ADHD, linking impulsivity to genes associated with 5-HT regulation (Oades et al., 2008). Recent studies have suggested the linkage between 5-HT receptor genes and the manifestation of ADHD, with 5-HT_2A_ and 5-HT_1B_ receptors in particular emerging as the most consistently implicated subtypes. Quist et al. reported a significant over-transmission of the 452Tyr variant of the *HTR2A* gene in children with ADHD, suggesting a potential genetic contribution of 5-HTergic pathways to the disorder (Quist et al., 2000). One study conducted a family association analysis to investigate polymorphisms in the *HTR1B* (encoding the 5-HT1B receptor) and *SLC6A4* genes. While most individual single-nucleotide polymorphisms (SNPs) showed no direct association with ADHD, the study revealed preferential maternal transmission of the C861 allele of *HTR1B*, as well as the STin2.12 allele of *SLC6A4*, to children with ADHD. Moreover, a significant gene-gene interaction between these loci was identified. These findings point to the importance of considering not just single genes, but how they interact, and how patterns of inheritance might shape vulnerability to ADHD (Banerjee, Banerjee, Chatterjee, Sinha, & Nandagopal, 2012). Further support for 5-HTergic involvement in ADHD comes from studies examining genes involved in 5-HT synthesis. A study by Baehne et al. demonstrated that polymorphisms in the TPH2 gene, which encodes the rate-limiting enzyme in serotonin biosynthesis, are associated with altered response inhibition and prefrontal cortex function in both ADHD patients and healthy individuals. Specifically, ADHD-associated risk alleles were linked to reduced NoGo-anteriorization, a neurophysiological marker of impaired response control, suggesting that 5-HTergic genetic variation can directly influence neural mechanisms underlying behavioral inhibition, a core deficit in ADHD (Baehne et al., 2009). Supporting these findings, preclinical research in mice has demonstrated a causal link between 5-HT₁_B_ receptor signaling and impulsive behavior. Nautiyal and colleagues showed that deletion of the *Htr1b* gene leads to heightened impulsive action. Restoration of 5-HT₁_B_ receptor expression in adulthood normalized these deficits, indicating a direct role for 5-HT₁_B_ signaling in the control of impulsivity (Nautiyal et al., 2017). In contrast, a study examining two 5-HT_2A_ gene (*HTR2A)* polymorphisms found no significant differences in genotype frequencies between individuals with ADHD and controls. Additionally, these polymorphisms showed no association with the clinical course or outcome of ADHD during adolescence (Guney, 2014). These conflicting results highlight the complexity of 5-HTergic involvement in ADHD and the need for further investigation to clarify these mechanisms.

Current pharmacological approaches to ADHD predominantly target catecholaminergic pathways, with comparatively limited emphasis on 5-HTergic mechanisms. Psychostimulants such as methylphenidate and amphetamines the most commonly prescribed medications primarily act on dopaminergic and noradrenergic systems, with additional modulatory effects on 5-HTergic pathways (Jackson, Riley, & Overton, 2025). Although SSRIs are frequently used to manage comorbid anxiety and obsessive-compulsive symptoms, evidence for their efficacy in improving core ADHD symptoms is inconsistent and generally inferior to first-line stimulant treatments (Hilo, Hale, Pham, & Khanfar, 2025). Further research is needed to clarify whether targeted 5-HTergic modulation could offer meaningful benefits for specific ADHD phenotypes.

**Fig. 5:**
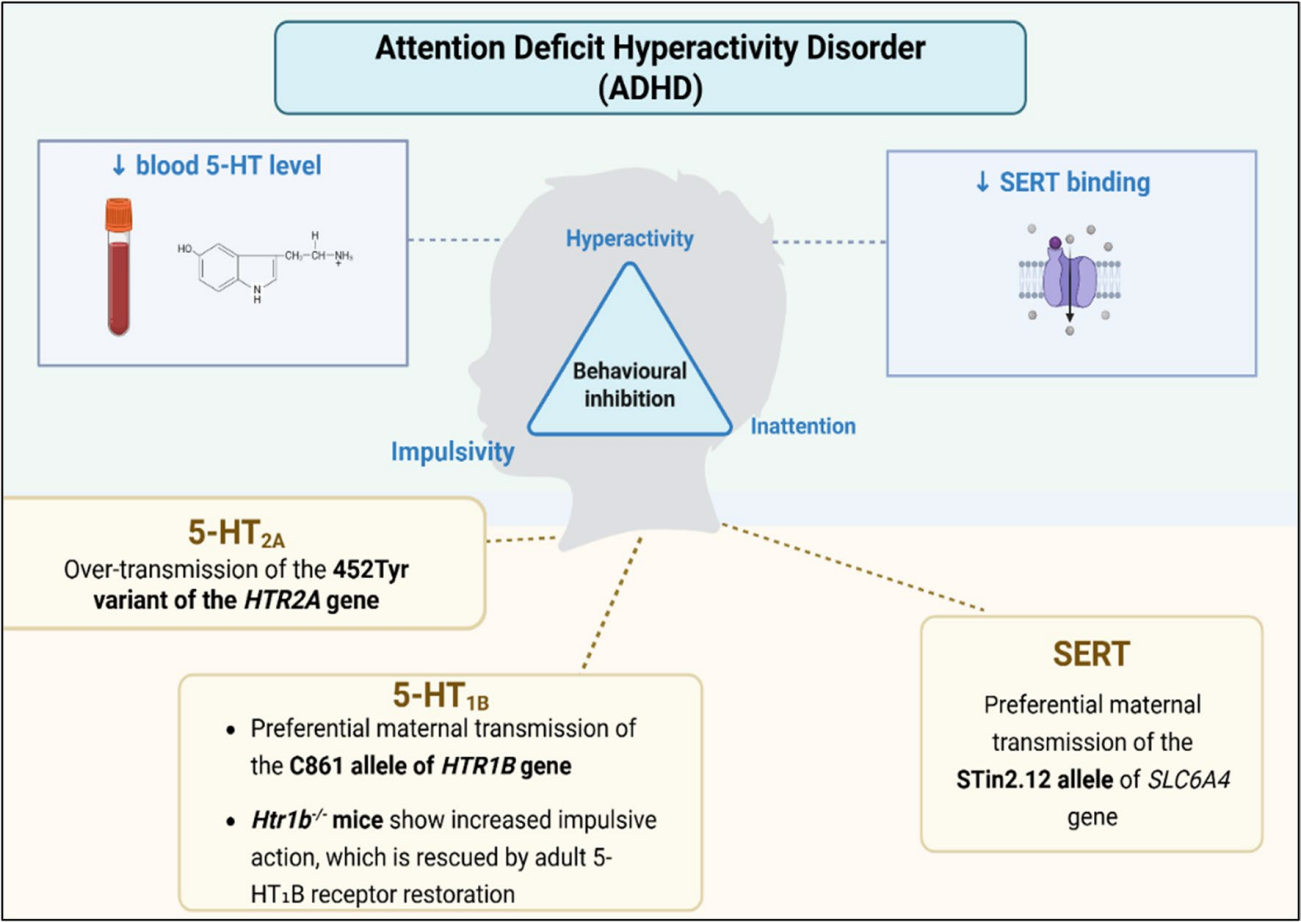
A diagram summarizing the key clinical features and the current findings on the involvement of the 5-HTergic system in ADHD. ADHD is characterized by a core deficit of behavioral inhibition, along with clinical features including hyperactivity, impulsivity and inattention. Studies have found that lowered 5-HT in the blood of children with ADHD, as well as reduced SERT binding, among ADHD patients. Genes encoding 5-HT receptors and SERT are implicated in ASD pathophysiology, with findings including over-transmission of the *HTR2A* 452Tyr variant and preferential maternal transmission of alleles in the C861 allele of the *HTR1B* gene and the STin2.12 allele of the *SLC6A4* gene. *Htr1b* knockout mice show increased impulsive action, and adult restoration of 5-HT₁_B_ receptor expression normalizes this behavior (ADHD: Attention Deficient Hyperactivity Disorder; SERT: serotonin transporter; *SLC6A4*: solute carrier family 6 member 4; *HTR1B*: 5-Hydroxytryptamine Receptor 1B; *HTR2A*: 5-Hydroxytryptamine Receptor 2 A; 5-HT_2A_: serotonin receptor 2 A; 5-HT_1B_: serotonin receptor 1B). Created in BioRender

In summary, while there is evidence supporting a role for the 5-HTergic system in ADHD, such as reduced peripheral 5-HT, altered SERT binding, and associations with 5-HT receptor variants, the findings are mixed. Future studies should prioritize comprehensive large-scale genetic analyses and consider gene-gene and gene-environment interactions to better elucidate the 5-HTergic contribution to ADHD pathology (Fig. 5).

## Conclusion

5-HT plays a critical role in brain development by regulating processes such as synaptic plasticity, neurogenesis, and the balance between excitatory and inhibitory signaling. Dysregulation of 5-HT signaling has been implicated in several neurodevelopmental disorders, including ASD, FXS, and ADHD. In ASD, altered 5-HT signaling and receptor activity have been linked to abnormal brain development and behavior, highlighting specific receptor subtypes, such as 5-HT_2B_, 5-HT_4_, and 5-HT_1A_, as potential therapeutic targets. In FXS, modulation of 5-HT_7_ receptor activity has shown promise in restoring synaptic plasticity. Evidence for 5-HT involvement in ADHD remains more heterogeneous, although altered 5-HT levels and genetic variation in receptor and transporter genes suggest a contributory role.

Importantly, the effects of 5-HT dysregulation are highly dependent on developmental timing. Early alterations in maternal and placental signaling may influence fetal brain development, while disruptions during embryonic and postnatal stages can affect circuit formation, synaptic refinement, and long-term behavioral outcomes. While these findings highlight the broad impact of 5-HT across development, several limitations remain. Much of the current evidence is derived from animal models, and the extent to which these findings translate to human neurodevelopment remains uncertain. In addition, the heterogeneity of neurodevelopmental disorders and variability in 5-HT findings across studies complicate the identification of consistent mechanisms. Future research should focus on integrating longitudinal human studies with mechanistic approaches to better define stage-specific roles of 5-HT signaling. In particular, identifying reliable biomarkers and clarifying critical developmental windows for intervention will be essential for translating these insights into effective therapeutic strategies.

## Data Availability

No datasets were generated or analysed during the current study.
